# IRES Mutation Confers a Replicative Advantage to SVA by Enhancing Translation Efficiency

**DOI:** 10.1155/tbed/7653487

**Published:** 2026-08-26

**Authors:** Tao Li, Wen Dang, Weiwei Li, Xiaodong Qin, Fan Xu, Hong Tian, Fan Yang, Yanyan Chang, Haixue Zheng

**Affiliations:** ^1^ State Key Laboratory for Animal Disease Control and Prevention, Lanzhou Veterinary Research Institute, Chinese Academy of Agricultural Sciences, College of Veterinary Medicine, Lanzhou University, Lanzhou 730046, China, lzu.edu.cn

**Keywords:** antiviral innate immunity, IRES, serial passage, SVA

## Abstract

The internal ribosome entry site (IRES) is a cis‐acting element found in certain RNA viruses. In virus‐infected cells, the Senecavirus A (SVA) IRES can directly recruit the small ribosomal subunit to an internal initiation codon on the mRNA, enabling translation initiation independently of both the 5^′^ cap structure of the viral genome and the host cell eukaryotic initiation factor 4F (eIF4F). As a small RNA virus, SVA frequently accumulates genetic mutations during transmission. This study aimed to characterize the mutational and evolutionary patterns of the SVA genome during its transmission within host cells exhibiting enhanced innate immunity. SVA was serially passaged for 80 generations in preactivated 3D4/21 cells that had been established in an antiviral innate immune state. During serial passaging, two stable single‐nucleotide mutations were consistently identified in the IRES region of the viral genomic RNA from passages 60–80: a uridine (U) insertion at genomic position 109, and a guanine (G)‐to‐adenine (A) substitution at position 265. Furthermore, the rescued SVA mutants harboring these mutations exhibited significantly higher replication titers than the parental strain. Notably, our results indicated that the mutant virus was unable to evade host antiviral innate immunity, whereas it significantly enhanced the translational activity of the SVA IRES. Collectively, these findings provide a foundation for understanding how single nucleotide polymorphisms (SNPs) in the 5^′^ untranslated region (UTR) region influence viral IRES activity and the replication capacity of recombinant viruses.

## 1. Introduction

Senecavirus A (SVA) is a recently emerged pathogen that causes clinical signs in pigs indistinguishable from those caused by foot‐and‐mouth disease virus (FMDV) and swine vesicular disease virus (SVDV) [1]. It can lead to abortion in sows and acute mortality in piglets [2]. To date, clinical outbreaks of SVA have been officially reported in several countries, including the United States [3], Canada [4], Brazil [5], Chile [6], Colombia [7], and Thailand [8]. Notably, clinical cases of SVA have also been reported in various provinces across China [9, 10]. Importantly, SVA nucleic acid has been detected in mice [4], mink [11], buffaloes [12], houseflies [13], and culicoides [14], suggesting that SVA is no longer restricted to swine intraspecies transmission and is undergoing continuous evolution to facilitate cross‐species transmission [15].

The SVA genome contains a single open reading frame (ORF) flanked by a 5^′^ untranslated region (UTR) and a 3^′^ poly(A) tail. The ORF encodes a polyprotein that is cleaved into four structural proteins (VP4, VP2, VP3, and VP1) and eight nonstructural proteins (L, 2A, 2B, 2C, 3A, 3B, 3C, and 3D) [16]. Notably, the 5^′^ UTR of the SVA genome, together with 55 nucleotides of the viral coding sequence, forms a functionally competent internal ribosome entry site (IRES) [17]. Similar to all members of the *Picornaviridae* family, the SVA IRES can directly recruit the small ribosomal subunit to an internal initiation codon through various RNA–RNA and RNA‐protein interactions, allowing translation initiation independently of a 5^′^ cap structure [18]. Furthermore, the SVA IRES functions independently of the eukaryotic initiation factor 4F (eIF4F) in virus‐infected cells [19, 20]. Crucially, IRES‐driven translation can continue even when the predominant cap‐dependent translation pathway is inhibited [21]. Importantly, the SVA IRES is essential for virus recovery [22]. RNA pseudoknots are prevalent structural elements found in the genomes of most RNA viruses. The IRES of HCV‐like viruses forms two hairpin structures, known as domain II and domain III [23]. Moreover, the 5^′^ UTR is structurally and functionally conserved with the fact that the 5^′^ UTR of all picornaviruses evolves independently from the rest part of the genome and can be recombinant with other RNA viruses [24].

As a member of the *Picornaviridae* family, SVA relies on RNA‐dependent RNA polymerase (RdRp) for replicating its RNA genome, which lacks robust proofreading capability [25]. This inherent characteristic makes SVA highly prone to genetic mutations, potentially conferring a selective advantage to the virus during epidemic spread [26, 27]. Additionally, innate immunity serves as the primary immune barrier against pathogen invasion [28]. When viruses infect cells in vitro, innate immunity provides the sole major immune protection for host cells to defend against viral attack, as adaptive immunity is absent [29, 30]. Accordingly, selective pressure imposed by innate immunity acts as a key driver of viral mutations. Serial passaging [31], a classical experimental approach, is widely used to simulate and investigate the genetic variation and evolutionary dynamics of RNA viruses in vitro [32, 33]. Although previous studies have characterized the genomic mutations of SVA following serial passaging [34, 35], they did not actively enhance cellular innate immune pressure. Furthermore, the specific mutation trends within the IRES region and the phenotypic changes of the virus under enhanced immune stress have not been systematically elucidated. This constitutes a critical knowledge gap in fundamental SVA virology, necessitating further investigation.

In this study, we characterized the mutational and evolutionary signatures of the SVA genome during its propagation in host cells exhibiting enhanced innate immunity. SVA underwent 80 serial passages in preactivated 3D4/21 cells with sustained antiviral innate‐immune activation. Next‐generation sequencing (NGS) analysis demonstrated that two stable single‐nucleotide mutations were consistently present in the IRES region of the viral genomic RNA from passages 60–80. Interestingly, compared to the parental strain, the rescued mutant SVA harboring these mutations exhibited significantly increased replication titers. Furthermore, functional assays revealed that the mutants could not evade host antiviral innate immunity, whereas the IRES‐mediated translation efficiency was markedly enhanced. Collectively, our results suggest that mutations in the IRES region confer SVA with superior intracellular replication capacity by increasing the translation efficiency of viral proteins rather than by enabling evasion of host antiviral innate immunity.

## 2. Materials and Methods

### 2.1. Cells

3D4/21 (ATCC CRL‐2843) cells were cultured in RPMI‐1640 medium (Gibco, China) supplemented with 10% (v/v) fetal bovine serum (FBS; ExCell Bio, China) and maintained at 37°C under 5% CO_2_ atmosphere. IBRS‐2 (Cellosaurus CVCL_4528) and HEK‐293T (ATCC CRL‐3216) cells were cultured in DMEM (Gibco, China) supplemented with 10% (v/v) FBS (ExCell Bio, China) under the same incubation conditions. All cell lines used in this study were preserved and routinely maintained in our laboratory.

### 2.2. Viruses

The SVA‐WT isolate CH‐FJ‐2017 (GenBank: KY74510) was previously isolated and characterized by our laboratory. The SVA mutants were rescued and harvested through the reverse genetic system.

### 2.3. Conditioned Medium Preparation

3D4/21 (ATCC CRL‐2843) cells were cultured in T25 flasks with complete growth medium (10% FBS). After infection with SVA inoculum (MOI = 0.1, 2% FBS) for 2 h, the inoculum was removed and replaced with fresh complete growth medium. Following an additional 36 h of incubation, the supernatant was collected, clarified by centrifugation at 1500 rpm for 10 min, and filtered through a 0.2 μm membrane. Viral inactivation was performed using ultraviolet (UV) irradiation at 253.7 nm for 2 h. Complete inactivation was confirmed by the absence of cytopathic effect (CPE) in IBRS‐2 cells inoculated with a 1:1 dilution of the treated supernatant. The resulting conditioned medium (CCM) was then aliquoted and stored at –80°C for short‐term use.

### 2.4. Virus Passage and Whole Genome Sequencing

SVA‐WT (CH‐FJ‐2017) was used to infect 3D4/21 cells at MOI of 0.1 in a T25 flask. After 1 h of incubation, the viral inoculum was discarded, and the cells were overlaid with fresh culture medium. The supernatant was collected after an additional 36 h of culture, and viral RNA copy numbers were quantified via RT‐qPCR titration. The harvested virus stock was then used to reinfect naive 3D4/21 cells. This serial passaging procedure was repeated for a total of 80 passages. Finally, viral RNA was extracted and subjected to NGS analysis at Shanghai Tanpu Biotechnology Co., Ltd. (Shanghai, China).

### 2.5. Dual‐Luciferase Reporter Assay

To evaluate and compare the IRES‐mediated translation activities of SVA‐WT and SVA mutants, we constructed a bicistronic luciferase reporter plasmid containing an SVA‐IRES element positioned between the Renilla and Firefly luciferase genes. Cells were seeded in 96‐well plates and cotransfected with dual‐luciferase reporter plasmids, including pIFN‐β‐luc (50 ng), pISRE‐luc (50 ng), pNF‐κB‐luc (50 ng), IRES (50 ng), or an empty vector plasmid (50 ng), along with pRL‐TK (5 ng). CCM (1:1 dilution), Poly(I:C) (1 μg/mL), TNF‐α (10 ng/mL), IFN‐β (10 ng/mL), or complete culture medium were added to the transfected cells, followed by incubation for 24 h. Firefly luciferase (F‐Luc) and Renilla luciferase (R‐Luc) activities were quantified with the dual‐luciferase reporter assay system (Promega) using a GloMax 20/20 luminometer.

### 2.6. Rescue, Detection, and Characterization of SVA Mutants

Infectious cDNA clones of SVA mutants carrying the designated mutations were generated. Briefly, IBRS‐2 cells were seeded in T‐25 flasks and transfected with 2.5 μg of infectious cDNA plasmid using Lipofectamine 3000 (Gibco,China). At 36 h post‐transfection, the culture underwent one freeze–thaw cycle and was then clarified by centrifugation. The supernatant was transferred to naive IBRS‐2 cells and passaged until clear CPEs were observed. The SVA mutants were subsequently amplified through five additional passages in IBRS‐2 cells to generate viral stocks and evaluate genetic stability and potential reversion events. Viral RNA was extracted from the harvested virus, and Sanger sequencing was performed by Sangon Biotech (Shanghai) Co., Ltd. (Shanghai, China) to confirm the presence of the intended mutations and assess genomic integrity.

### 2.7. Growth Curves of SVA Mutants

SVA was used to infect cells cultured in 12‐well plates with a medium supplemented with 2% (v/v) FBS for 2 h. After infection, the viral inoculum was removed, and each well was overlaid with 2 mL of fresh complete growth medium containing 10% (v/v) FBS. The cell culture supernatants were harvested at 12, 24, and 36 h postinfection. Viral RNA copy numbers in the culture supernatants were quantified using the EVO M‐MLV One‐Step RT‐PCR Kit (Accurate Biology). Furthermore, viral titers in the cell culture supernatants were determined via the TCID_50_ assay. Briefly, IBRS‐2 cells were seeded into 96‐well plates and incubated overnight. The collected supernatants were serially diluted 10‐fold, and each dilution was added to eight replicate wells at a volume of 100 μL per well. After 24 h of incubation, CPEs were observed and recorded, and viral titers were calculated by the Reed–Muench formula.

### 2.8. qRT‐PCR

Cells were washed twice with PBS and lysed in lysis buffer at room temperature with gentle shaking. Total RNA was extracted using the SteadyPure Universal RNA Extraction Kit (Accurate Biology). After RNA quantification, equal amounts of RNA were subjected to reverse transcription using the PrimeScript RT Master Mix (Takara). Next, quantitative real‐time PCR (qRT‐PCR) was performed using the TB Green Premix Ex Taq (Takara). All primer sequences utilized in this study are summarized in Table 1.

**Table 1:** PCR primer.

Primers	Sequences (5′‐3′)
GAPDH	Forward: 5′‐AGTGGACATTGTCGCCATCA‐3′
	Reverse: 5′‐TGACTGTGCCGTGGAACTTG‐3′
RIG‐I	Forward: 5′‐TCTGGAGCAACGTCTGACAC‐3′
	Reverse: 5′‐GTCAGTCCAATGACCTGGGG‐3′
IFN‐β	Forward: 5′‐GATGGGCAGATGGATGACCT‐3′
	Reverse: 5′‐AATGGTCATGTCTCCCCTGG‐3′
IFIT1	Forward: 5′‐TCCGACACGCAGTCAAGTTT‐3′
	Reverse: 5′‐TGTAGCAAAGCCCTGTCTGG‐3′
IFIT2	Forward: 5′‐GCACAGCAATCATGAGTGAGAC‐3′
	Reverse: 5′‐GCTTGCCTCAGAGGGTCAAT‐3′
IFIT3	Forward: 5′‐AGGAGGCCATTGAGTTGAGC‐3′
	Reverse: 5′‐CATCTGCACCCTGGTCCATT‐3′
CCL4	Forward: 5′‐TTCACATACACCGTGCGGAA‐3′
	Reverse: 5′‐ACTCCTGGACCCAGTCATCA‐3′
CCL5	Forward: 5′‐CCCCATATGCCTCGGACAC‐3′
	Reverse: 5′‐AATATTCCTGGAGGTGGGCG‐3′
OAS1	Forward: 5′‐CAGACTCCACGACAGGAGAA‐3′
	Reverse: 5′‐TTCCGGGTTGGGTTTGTAGC‐3′
MX2	Forward: 5′‐GTCATCGGGGACCAGAGTTC‐3′
	Reverse: 5′‐CTCCACTTTGCGGTAGCTGA‐3′
ISG15	Forward: 5′‐CCGGCAATGTGCTTCAGGAT‐3′
	Reverse: 5′‐CCTTGTCGTTCCTCACCAGG‐3′

### 2.9. Transcriptome Sequencing

Cells were cultured in 6‐well plates. Three wells were designated as the negative control (NC) and incubated with complete culture medium only. Each experimental group was set up with three biological replicate wells. All operations were carried out on ice to reduce RNA degradation. Briefly, cells were lysed using TRIzol reagent, and the resulting lysates were collected and immediately flash‐frozen in liquid nitrogen. The frozen lysates were then shipped to OE Biotech Co., Ltd. (Shanghai, China) for transcriptome sequencing and subsequent bioinformatic analysis. Differentially expressed genes were screened from the transcriptomic sequencing data based on the thresholds log_2_ fold change >1 and *q*‐value < 0.05 for follow‐up analysis.

## 3. Results

### 3.1. Antiviral Innate Immunity in Cells Preactivated by Conditioned Medium

**Figure 1:**
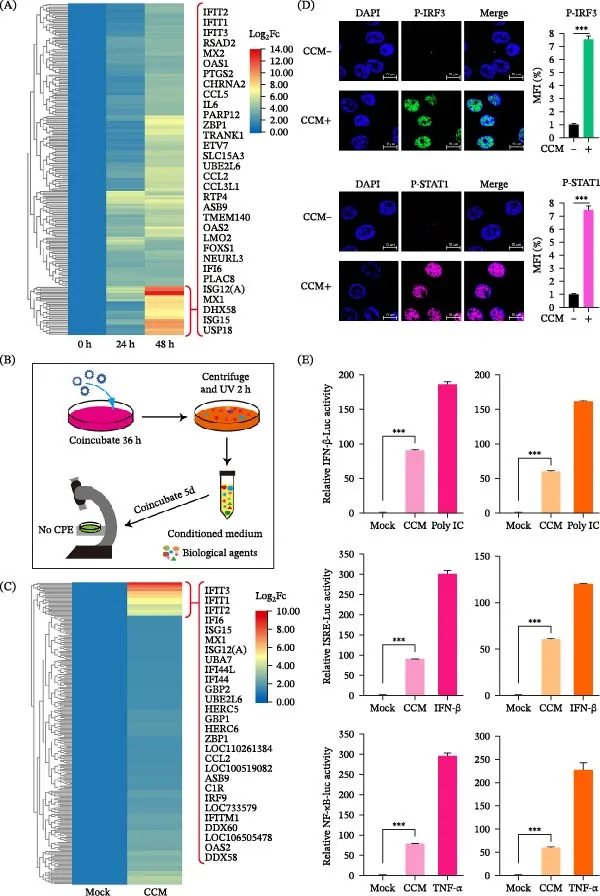
Conditioned medium enhances cellular antiviral innate immune responses. (A) The transcriptional levels in 3D4/21(CRL‐2843) cells after infection with SVA. (B) The procedure for preparing conditioned medium. (C) The transcriptional levels in 3D4/21(CRL‐2843) cells after incubation with CCM for 18 h. (D) The expression levels and nuclear translocation of phosphorylated IRF3 (P‐IRF3) and phosphorylated STAT1 (P‐STAT1) in 3D4/21 (CRL‐2843) cells following 18 h incubation with CCM were assessed by immunofluorescence assay (IFA). Cells were incubated with CCM for 18 h. Following fixation, the cells were incubated overnight (12 h) with primary antibodies against P‐IRF3 and P‐STAT1, then incubated with appropriate secondary antibodies for 1 h. Fluorescence signals were subsequently visualized using a Laser Scanning Confocal Microscope (Zeiss LSM980, Carl Zeiss), and fluorescence intensity was quantitatively analyzed using Image J. (E) The immune activation status of cells after 18 h of incubation with CCM was assessed using dual‐luciferase reporter assay. Cells (CRL‐2843, left column; HEK‐293T, right column) were seeded in 96‐well plates and cotransfected with dual‐luciferase reporter plasmids, including pIFN‐β‐luc (50 ng), pISRE‐luc (50 ng), pNF‐κB‐luc (50 ng), or an empty vector plasmid (50 ng), along with pRL‐TK (5 ng). CCM (1:1 dilution), Poly(I:C) (1 μg/mL), TNF‐α (10 ng/mL), or IFN‐β (10 ng/mL) were added to the transfected cells, followed by incubation for 24 h, F‐Luc and R‐Luc were measured. All experiments were repeated three times with consistent results. Student’s *t*‐test:  ^∗^ *p* < 0.05,  ^∗∗^ *p* < 0.01,  ^∗∗∗^ *p* < 0.001.

The cellular antiviral innate immune response serves as the host’s first line of defense against viral infections [36]. Key components of this response include interferons (IFNs), interferon‐stimulated genes (ISGs), proinflammatory cytokines, and other associated molecules [37, 38], which work in concert to establish a robust antiviral state within cells. Previous studies have demonstrated that 3D4/21 (CRL‐2843) cells are highly susceptible to SVA infection and can induce a robust innate immune response, including both IFN and inflammatory signaling pathways [39, 40]. Here, we further characterize the antiviral innate immune response induced by SVA infection in 3D4/21 cells using transcriptome sequencing analysis (RNA‐seq). Our results demonstrate that SVA infection significantly upregulates the mRNA expression of key ISGs with potent antiviral activities, including IFIT1, IFIT2, IFIT3, MX1, MX2, OAS1, OAS2, ZBP1, ISG12A, and ISG15. Additionally, the transcription of major proinflammatory mediators, such as IL6, CCL2, CCL3L1, and CCL5, is markedly enhanced. These findings indicate that SVA infection triggers a robust and coordinated innate immune response in 3D4/21 cells, leading to the activation of antiviral effectors, including type I IFNs, ISGs, and proinflammatory cytokines (Figure 1A).

To further investigate the functional components of this response, CCM was collected from SVA‐infected 3D4/21 cells. As shown in Figure 1B, cells were infected with SVA for 2 h, after which the viral inoculum was removed. The cells were then washed three times with PBS to eliminate residual virus, followed by the addition of fresh complete growth medium. After an additional 36 h of incubation, the cells were subjected to one freeze–thaw cycle to release intracellular factors, and the culture supernatant was subsequently harvested. To inactivate any remaining infectious viral particles, the supernatant was exposed to UV irradiation (253.7 nm) for 2 h. The CCM was mixed with fresh complete growth medium at a 1:1 ratio, and the mixture was inoculated onto IBRS‐2 cells—an established, highly permissive cell line for SVA—followed by incubation for 5 days. No CPE was observed during this period, suggesting the absence of viable virus. The resulting CCM was then aliquoted and stored at −80°C for short‐term use (Figure 1B).

Subsequently, CCM was mixed with complete culture medium at a 1:1 ratio and added to naive 3D4/21 cells, followed by an 18‐h incubation. Transcriptomic sequencing analysis of these cells showed that preincubation with CCM for 18 h robustly induced an innate antiviral immune response in uninfected cells, as evidenced by significant upregulation in the transcriptional levels of antiviral ISGs, including IFIT1, IFIT2, IFIT3, IFI6, ISG15, MX1, ISG12A, ZBP1, IFITM1, and others (Figure 1C). Then, immunofluorescence assay (IFA) results revealed that in CCM‐stimulated cells, phosphorylated IRF3 (P‐IRF3) was upregulated and translocated into the nucleus—a crucial step for IFN induction. Similarly, phosphorylated STAT1 (P‐STAT1) levels were increased and accumulated in the nucleus, which is essential for the transcriptional activation of ISGs (Figure 1D). To further assess the immune activation status in CCM‐stimulated cells, dual‐luciferase reporter assays driven by the IFN‐β promoter, NF‐κB promoter, and ISRE were performed in both 3D4/21 and HEK293T cells following CCM stimulation. These assays confirmed that CCM exerts robust immunostimulatory activity (Figure 1E). These findings demonstrate that uninfected 3D4/21 cells, when stimulated with CCM, can establish an antiviral innate immune state similar to that observed in virus‐infected cells. Thus, CCM acts as a potent activator of antiviral innate immunity.

### 3.2. SVA Was Serially Passaged in 3D4/21 Cells and Acquired Mutations

**Figure 2:**
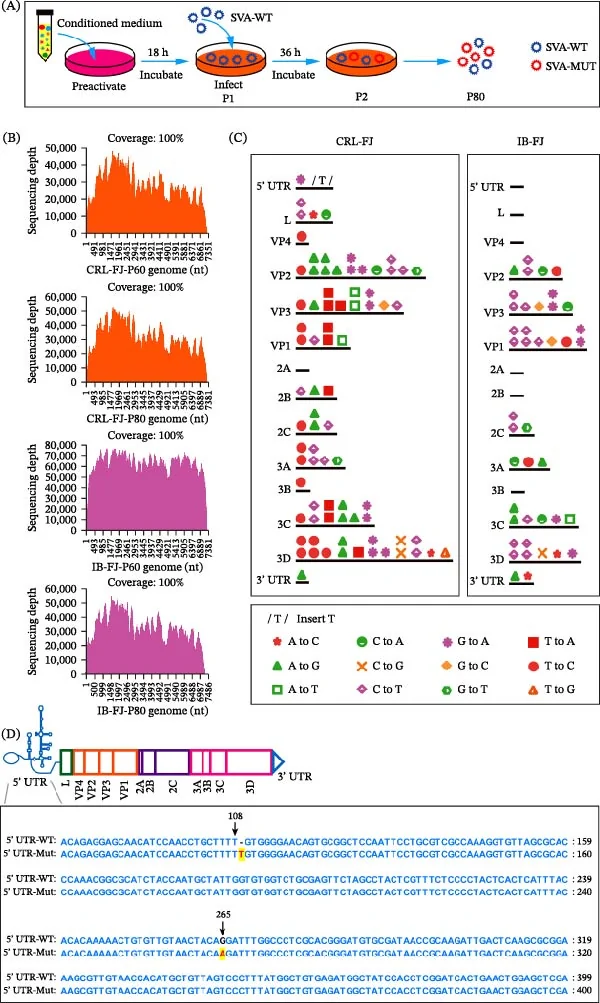
Serial passaging of viruses and whole‐genome next‐generation sequencing analysis. (A) Procedure for serial passaging of SVA in CRL‐2843 cells with antiviral innate immunity preactivated by CCM. (B) Sequencing coverage and depth. (C) Comparison of stably inherited nucleotide mutations in the P60–P80 genomic regions of CRL‐FJ and IB‐FJ. In the figure, distinct symbols represent different types of nucleotide substitutions, and the frequency of each symbol corresponds to the number of occurrences of that specific mutation type. (D) Two distinct mutations occurred in the 5^′^‐UTR of CRL‐FJ: 108U109 and G265A.

SVA belongs to the *Picornaviridae* family. Like other RNA viruses, it replicates using RdRp, which lacks proofreading capability. Consequently, this leads to frequent mutations and genetic recombination during transmission, making the virus prone to generating diverse strains [41]. To explore the mutations and evolutionary characteristics of SVA during its propagation in cells with enhanced innate immune responses, 3D4/21 cells (CRL‐2843) were preincubated with CCM for 18 h to induce an antiviral innate immune response prior to infection. The cells were then infected with SVA at MOI of 0.1 and cultured for 36 h, after which progeny virus was collected from the supernatant. This serial passaging procedure was carried out for up to 80 generations (Figure 2A). Notably, IBRS‐2 cells are known to harbor a defective RIG‐I‐like receptor (RLR) signaling pathway and thus fail to elicit an effective innate immune response upon SVA infection [42]. As such, IBRS‐2 cells without CCM pretreatment were used as a control group, in contrast to CCM‐pretreated 3D4/21 cells. SVA was passaged in parallel in these two distinct cellular environments—designated IB‐FJ (passaged in IBRS‐2 cells without CCM prestimulation) and CRL‐FJ (passaged in CCM‐prestimulated 3D4/21 cells)—enabling a comparative analysis of viral mutational patterns and evolutionary dynamics under low versus high innate immune pressure. After 80 serial passages, viral RNA was extracted from progeny viruses at passages P0, P20, P40, P60, and P80 and subjected to NGS analysis performed by Shanghai Tanpu Biotechnology Co., Ltd. (Shanghai, China).

The sequencing results showed a progressive increase in the number of single‐nucleotide mutations in the SVA genome with increasing passage number. Notably, stably inherited mutations predominantly emerged between passages 60 and 80, indicating that the formation of stable genetic variants under antiviral innate immune pressure probably depends on a threshold level of accumulated selection. As shown in Figure 2B, the whole‐genome NGS data from the P60 and P80 samples exhibited high coverage and sequencing depth, demonstrating high reliability and providing a robust foundation for downstream analysis (Figure 2B). As shown in Figure 2C, comparative analysis of the two passage lineages—IB‐FJ and CRL‐FJ—revealed that CRL‐FJ accumulated significantly more nucleotide substitutions than IB‐FJ in the VP2, VP3, 3C, and 3D genomic regions during passages 60–80. Moreover, mutations were detected in additional regions in CRL‐FJ, including the 5^′^ UTR, L, VP4, 2B, and 3B, whereas no such mutations were observed in these regions in IB‐FJ over the same passage interval (Figure 2C). Specifically, the mutations shown above are based on NGS data, characterized by single nucleotide polymorphisms (SNPs) with variant allele frequencies (VAFs) above 99.9%.

Previous studies have shown that SVA can regulate translation through IRESs, thereby facilitating viral replication. Correspondingly, increased IRES efficiency can elevate viral translational efficiency [43]. The SVA IRES is classified as an HCV‐like IRES. It folds into a well‐characterized secondary structure consisting of two major hairpin domains (domain II and domain III) and two pseudoknots (PKS‐I and PKS‐II). All these structural motifs are proven indispensable for SVA translation and replication [44]. Specifically, the basal segment of domain III mainly mediates the initial recruitment of the 40S ribosomal subunit, and nucleotide mutations within the PKS structures affect viral rescue from cDNA and alter viral in vitro growth kinetics [18, 23]. However, to date, no study has elucidated the mutation patterns of the SVA IRES under enhanced innate immune pressure or the impact of these mutations on viral rescue and in vitro growth kinetics. Importantly, our experimental results showed that the CRL‐FJ virus exhibited two stable nucleotide mutations in the IRES, whereas the IB‐FJ virus showed no mutations in this region. We speculate that these two nucleotide mutations in the CRL‐FJ virus may have significant effects on the virus. Accordingly, this study primarily investigates these consecutive mutations, while other nucleotide mutations in the viral genome will be examined in future research studies.

As shown in Figure 2C, two stable mutations specifically emerged and persisted in the 5^′^ UTR of CRL‐FJ between passages 60 and 80 but were absent in IB‐FJ. Detailed sequence alignment (Figure 2D) revealed that these mutations consisted of a uridine insertion at genomic position 109 (designated IRES‐108U109) and a guanine‐to‐adenine substitution at position 265 (designated IRES‐G265A), both located within the IRES of the 5^′^ UTR.

### 3.3. Mutations Boost the Efficiency of Translation Initiation

**Figure 3:**
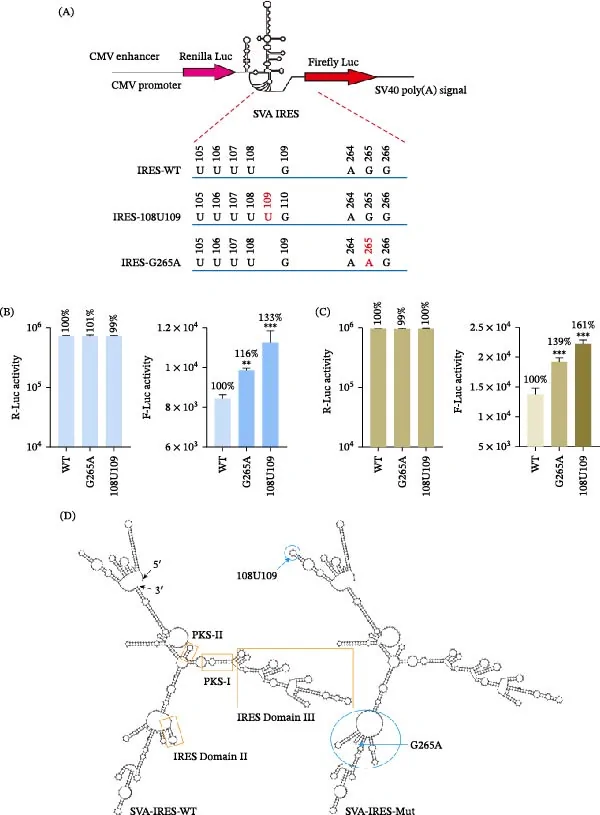
IRES‐dependent translation initiation assay. (A) Key components of the pCMV‐hR‐Luc‐IRES‐F‐Luc plasmid, including the positions and types of introduced mutations. (B, C) The pCMV‐hR‐Luc‐IRES‐F‐Luc plasmid was transfected into 3D4/21 cells (B) and HEK293T cells (C), Following 24 h of incubation, cells were lysed completely on ice. R‐Luc and F‐Luc activities were measured using a Dual‐Luciferase Reporter Assay System. (D) The secondary structures of both the wild‐type and mutant SVA IRES were predicted and modeled using the RNAfold web server (http://rna.tbi.univie.ac.at). In the structural diagrams, the blue circle highlights the region affected by the mutation, the red nucleotides indicate the specific sites of the mutated bases, and the yellow box marks the regions corresponding to the key structural domains of the IRES reported in prior literature. All experiments were repeated three times with consistent results. Student’s *t*‐test:  ^∗^ *p* < 0.05,  ^∗∗^ *p* < 0.01,  ^∗∗∗^ *p* < 0.001.

The 5^′^ UTR of SVA constitutes the core component of the IRES. Similar to the IRES of HCV, it is a cis‐acting element that can recruit the small ribosomal subunit to an internal initiation codon on the mRNA, thereby enabling cap‐independent translation initiation in infected cells [45, 46]. To investigate whether mutations in the 5^′^ UTR affect the activity of IRES in initiating translation, we constructed the pCMV‐hRluc‐IRES‐Fluc plasmid, as shown in Figure 3A; IRES activity does not influence the translation of the upstream hRluc gene but modulates the translation efficiency of the downstream Fluc gene (Figure 3A). The plasmid was transfected into 3D4/21 cells. After 5 h of incubation, the culture medium was replaced with fresh complete growth medium. Following an additional 24 h of incubation, cells were lysed completely on ice. Subsequently, R‐Luc and F‐Luc activities were measured using a Dual‐Luciferase Reporter Assay System. The results showed that neither the wild‐type (WT) nor the mutant IRES affected R‐Luc activity. However, the IRES mutants significantly enhanced F‐Luc activity. Notably, the IRES‐108U109 mutant caused a significantly greater increase in F‐Luc activity compared to the IRES‐G265A mutant (Figure 3B). The same experiment and detection were performed in HEK293T cells, and the results were consistent (Figure 3C).

In order to initially reveal the mechanism of the change in translation initiation activity of IRES, the secondary structure of the WT and mutant IRES was predicted and modeled using the RNAfold web server. The results revealed that, compared to the WT IRES, the mutant IRES exhibited distinct conformational alterations at the mutation site (Figure 3D). The secondary structure of the IRES predicted in this study can be roughly classified into three major intricate stem‐loop domains, denoted as domains I, II, and III. By comparing the previously documented sequence and domain of the SVA IRES with our predicted models, we found that the previously reported SVA IRES domain II and PKS‐II only partially matched our predictions [18]; in contrast, domain III and PKS‐I were completely consistent with our predicted results [19, 22]. We compared the computationally predicted secondary structures of the IRES derived from WT and mutant (MUT) strains. The IRES‐108U109 mutation lies within domain I and causes nonsignificant structural perturbations. In contrast, the IRES‐G265A mutation lies within domain II and results in marked secondary structural alterations.

### 3.4. Mutations Enhance Viral Replication in Host Cells

**Figure 4:**
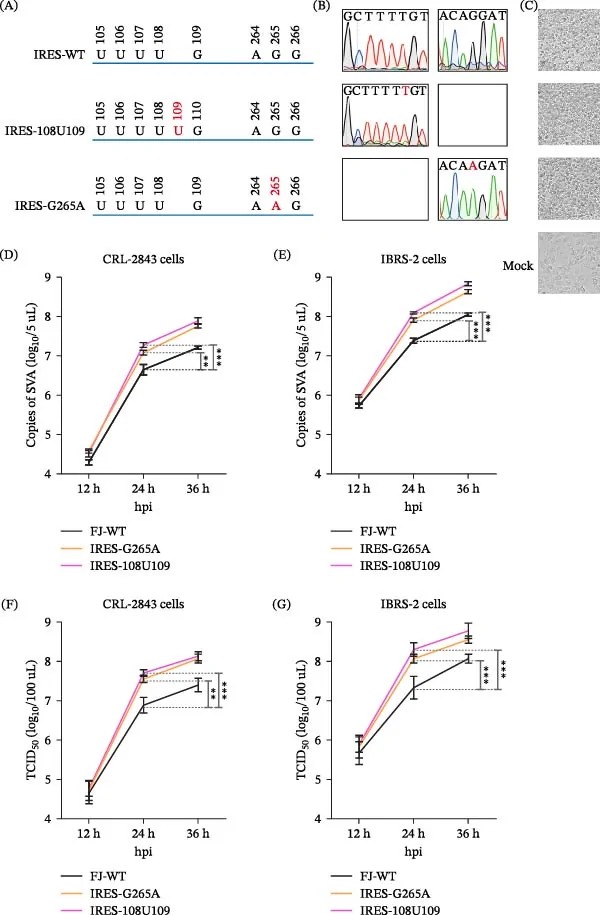
Recovery and growth kinetics assessment of SVA. (A) 5^′^ UTR mutations in the reverse genetics system for rescuing SVA were identified through sequencing. (B) 5^′^ UTR mutations in the rescued and recovered SVA strains were identified through sequencing. (C) CPE induced by the wild‐type and mutant viruses in 3D4/21 cells. (D, E) Viral RNA copy numbers of wild‐type and mutant SVA in culture supernatants from 3D4/21 cells (D) and IBRS‐2 cells (E) were quantified via RT‐qPCR at 12, 24, and 36 h postinfection. (F, G) Viral titers of wild‐type and mutant SVA in cell culture supernatants from 3D4/21 cells (F) and IBRS‐2 cells (G) were determined via the TCID_50_ assay at 12, 24, and 36 h postinfection. All experiments were repeated three times with consistent results. Student’s *t*‐test:  ^∗^ *p* < 0.05,  ^∗∗^ *p* < 0.01,  ^∗∗∗^ *p* < 0.001.

The reverse genetics system is a well‐established and reliable viral gene editing technology to rescue RNA viruses [47, 48]. To research the impact of mutations on viral replication, we employed a reverse genetics system to rescue both mutant and WT viruses, followed by whole‐genome sequencing of the rescued strains. Sequencing results confirmed that the intended nucleotide changes were accurately introduced into the viral genomes (Figure 4A,B). Then, 3D4/21 cells were infected with the rescued virus at MOI of 0.1. After 24 h, pronounced CPEs were observed, indicating that the rescued virus was fully capable of infecting host cells and inducing viral cytopathology (Figure 4C). Finally, we evaluated viral replication kinetics using one‐step growth curve analysis. 3D4/21 cells and IBRS‐2 cells were infected with the rescued virus at MOI of 0.1. After 2 h of attachment, the viral inoculum was removed, and the cells were washed three times with precooled PBS to eliminate unbound virions. Fresh complete culture medium was immediately added, and this time point was designated as 0 h postinfection (hpi). Subsequently, supernatants were collected at 12, 24, and 36 hpi, and viral RNA copies were quantified by RT‐qPCR. As shown in Figure 4D–E, both mutant viruses exhibited significantly enhanced growth and replication levels compared to the WT virus in cells. Notably, the IRES‐108U109 mutant displayed the highest level of growth and replication, followed by the IRES‐G265A mutant (Figure 4D,E). Importantly, TCID50 analysis was also performed on the collected supernatant, and the growth curve derived from viral titers showed strong consistency with that obtained from viral RNA copies (Figure 4F,G).

These findings indicate that mutations in the IRES region modulate its translation initiation activity, thereby affecting viral replication and growth dynamics in infected cells. Importantly, genomic data from 290 SVA isolates were downloaded from the NCBI database. We performed a comprehensive sequence alignment comparing the genome of the mutant virus with that of the WT virus as well as with those of previously reported SVA strains. Notably, the IRES‐108U109 mutant is absent from all other SVA strains, whereas the IRES‐G265A mutant is widely present in most other SVA strains. Table 2 summarizes the key findings. These findings suggest that the IRES‐G265A mutant is both beneficial to SVA and readily acquired through natural evolution, whereas the IRES‐108U109 mutant, although beneficial to SVA, is not easily achieved under natural evolutionary conditions (Table 2).

**Table 2:** Genome alignment results of SVA strains.

Virus strain	Source (country, year, host)	108U109	G265A
SVA‐FJ‐IRES‐WT	China, 2017, Pig	UG	AGG
SVA‐FJ‐IRES‐Mut	CRL‐FJ	UUG	AAG
KX751943.1	China, 2017, Pig	UG	AAG
MF189001.1	China, 2017, Pig	UG	AAG
MK039162.1	China, 2018, Pig	UG	AAG
MN781984.1	China, 2019, Pig	UG	AAG
KX223836.1	USA, 2015, Pig	UG	AAG
KU058183.1	USA, 2015, Pig	UG	AAG
MK357117.1	USA, 2019, Pig	UG	AAG
MN781984.1	USA, 2019, Pig	UG	AAG
KY486164.1	Canada, 2015, Pig	UG	AAG
PP836815.1	Canada, 2019, Pig	UG	AAG
KC667560.1	Canada, 2011, Pig	UG	AAG
PP836804.1	Canada, 2022, Pig	UG	AAG
KR063107.1	Brazil, 2015, Pig	UG	AAG
KR063108.1	Brazil, 2015, Pig	UG	AAG
KR063109.1	Brazil, 2015, Pig	UG	AAG
KX857728.1	Colombia, 2016, Pig	UG	AAG
MH704432.1	Viet Nam, 2018, Pig	UG	AAG

### 3.5. Mutant Viruses Do Not Acquire Replication Advantage by Evading Innate Immunity

**Figure 5:**
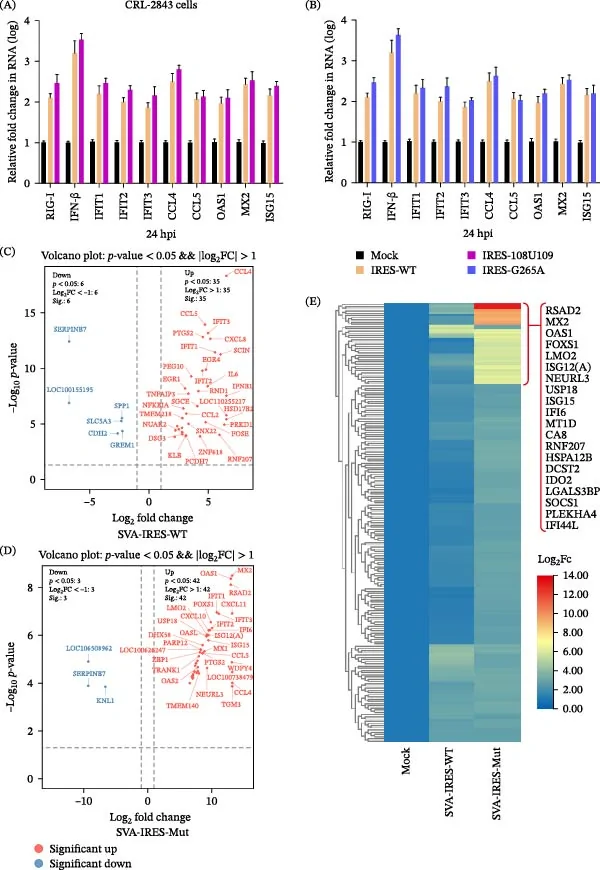
Detection of Viral Infection‐Induced Transcription of Antiviral Innate Immune Factors. 3D4/21 cells were infected with either the wild‐type virus (SVA‐IRES‐WT) or a single‐point mutation virus (IRES‐108U109, IRES‐ G265A), or a two‐point mutant virus (SVA‐IRES‐Mut), and cocultured for 24 h. (A, B) RT‐qPCR analysis of immune factor mRNA transcription levels in IRES‐108U109, IRES‐ G265A, and IRES‐WT infected cells. Results were compared with a mock control without virus infection. Special note: for graphical representation, the fold changes of the mock group were not log‐transformed, whereas those of the IRES‐WT, IRES‐108U109, and IRES‐G265A groups were expressed as log10 values. (C) Volcano plots based on transcriptome sequencing data showed upregulated and downregulated genes in 3D4/21 cells 24 h after infection with the wild‐type virus (SVA‐IRES‐WT). (D) Volcano plots based on transcriptome sequencing data showed upregulated and downregulated genes in 3D4/21 cells 24 h after infection with the two‐point mutant virus (SVA‐IRES‐Mut). (E) The clustering heatmap of transcriptome sequencing data revealed distinct gene expression profiles in 3D4/21 cells at 24 h postinfection with SVA‐IRES‐WT and SVA‐IRES‐Mut, highlighting divergent clustering patterns and marked upregulation of numerous genes. All experiments were repeated three times with consistent results.

Since the mutant virus was isolated following serial passaging under strong selective pressure from potent antiviral innate immunity, we aimed to further investigate the mechanism responsible for its replicative advantage. To assess whether the mutant virus evades host innate immune responses, 3D4/21 cells were synchronously infected with either the mutant or WT SVA at MOI of 0.1 for 24 h. Subsequently, cells were harvested, and total RNA was extracted for RT‐qPCR analysis. Compared to the uninfected mock control, infection with the IRES‐WT virus activated the antiviral innate immune response, resulting in a significant increase in the mRNA levels of representative innate immune effector genes (RIG‐I, IFN‐β, IFIT1/2/3, CCL4/5, OAS1, MX2, and ISG15). Notably, the IRES‐108U109 and IRES‐G265A mutants also induced robust antiviral innate immune responses, with the transcription levels of these effector genes being slightly higher than those observed in the IRES‐WT (Figure 5A,B).

Subsequently, we performed transcriptome sequencing on 3D4/21 cells synchronously infected with either the mutant or WT SVA at MOI 0.1 for 24 h. Volcano plot analysis revealed that infection with the SVA‐IRES‐WT significantly upregulated the transcription of IFN and ISG associated antiviral factors, including IFIT1, IFIT2, and IFIT3. Moreover, there was a marked induction of proinflammatory mediators, including IL‐6, CCL2, CCL4, CCL5, and CXCL8 (Figure 5C), which was consistent with the results shown in Figure 1A of this study. Similarly, infection with the SVA‐IRES‐Mut robustly activated the expression of antiviral innate immune genes (Figure 5D), including both IFN/ISG‐related factors (such as IFIT1, IFIT2, IFIT3, OAS1, OAS2, MX1, MX2, ISG12A, and ISG15) and inflammatory chemokines/cytokines (such as CCL4, CCL5, CXCL10, and CXCL11). To facilitate a direct comparison of the differences between the two groups, we further performed cluster‐based heatmap analysis. The results revealed that the transcriptional levels of key antiviral innate immune factors—including RSAD2, MX2, OAS1, ISG12, ISG15, and IFI6—were markedly higher in cells infected with the SVA‐IRES‐Mut than in those infected with the SVA‐IRES‐WT (Figure 5E). Additionally, genes involved in the regulation of cellular gene expression and immune modulation, such as FOXS1, LMO2, NEURL3, and USP18, also exhibited significantly elevated transcriptional levels in SVA‐IRES‐Mut‐infected cells compared to SVA‐IRES‐WT‐infected cells (Figure 5E).

These results indicate that the IRES‐108U109, IRES‐G265A, and SVA‐IRES‐Mut viruses are unable to evade the antiviral innate immune response in cells. Furthermore, due to their enhanced replication capacity in host cells, the mutated virus triggers a significantly stronger antiviral innate immune response compared to the SVA‐IRES‐WT virus. Collectively, our findings suggest that the significantly enhanced replication capacity of the Mut virus in host cells is not due to the evasion of the antiviral innate immune response. Instead, it results from increased IRES‐mediated translational activity, which promotes more efficient synthesis of viral proteins and thereby facilitates viral particle production.

## 4. Discussion

Translation of most cellular mRNA is initiated through a 5^′^cap‐dependent scanning mechanism. In contrast, a large portion of viral mRNAs employ IRES to initiate translation independently of the 5^′^cap structure [49]. The SVA genome is a nonsegmented, single‐stranded, positive‐sense RNA, featuring an ORF flanked by 5^′^ UTR and 3^′^ UTR [50].

The 5^′^ UTR of SVA, together with the initial coding sequence of the L protein, forms an IRES with full activity [17, 19]. According to reports, the SVA IRES is significantly similar to the HCV IRES, particularly in their secondary structures, which consist of two major stem‐loop domains, DII and DIII [20, 51]. As a result, studies on the HCV IRES have often served as a reference for understanding the SVA IRES. However, recent comparative analyses of secondary structures have revealed that the SVA IRES exhibits significant structural similarity to the CSFV IRES [17, 19]. Therefore, the structural and functional features of the SVA IRES are not yet fully understood and require further investigation.

Serial passaging is a fundamental experimental strategy for modeling and elucidating the patterns and mechanisms of viral mutation and evolution under controlled laboratory conditions [52, 53]. In a previous report, to characterize evolutionary patterns associated with CHIKV replication in mosquito cells, researchers performed serial passaging of the LR2006 strain in *Aedes albopictus* cells [54]. In another report, researchers serially passaged the highly pathogenic PDCoV strain CZ2020 for more than 100 generations. As the number of passages increased, CZ2020 progressively acquired enhanced in vitro fitness and became markedly attenuated [55]. Interestingly, researchers serially passaged a highly pathogenic African swine fever virus (ASFV) strain in Vero cells up to passage 30. During this process, the virus acquired mutations that altered its cellular tropism, allowing it to adapt efficiently to Vero cells [56].

In this study, SVA was serially passaged for 80 generations in 3D4/21 cells; these cells are capable of inducing effective antiviral innate immunity upon SVA infection. Prior to infection, these cells were pretreated with conditioned culture medium (CCM) to further enhance their antiviral innate immune state, thereby subjecting SVA to intense and sustained innate immune pressure. In contrast, IBRS‐2 cells—lacking functional innate immune signaling pathways and not preactivated with CCM—served as a control under minimal antiviral immune pressure. After 80 passages, NGS was used to analyze the evolutionary dynamics of the viral genome. Notably, between passages 60 and 80, two stable single‐nucleotide mutations emerged in the 5^′^ UTR of the CRL‐FJ strain, whereas no mutations were detected in the IB‐FJ strain. This strongly suggests that these mutations in CRL‐FJ were selected under strong antiviral innate immune pressure.

The dual‐luciferase reporter assay results revealed that both mutations can independently enhance the translation efficiency of proteins. Importantly, both mutations did not impair the rescue of infectious virus through reverse genetics system; rather, they provided significant replication advantages for viruses in cultured cells. Then, a comparison of computationally predicted IRES secondary structures between WT and MUT strains showed divergent outcomes: the IRES‐108U109 mutation within domain I caused minimal structural changes, whereas the IRES‐G265A mutation in domain II induced profound secondary structural rearrangements. Previous studies have shown that base mutations in the SVA IRES domain can affect the rescue of SVA viruses and the growth kinetics of mutated viruses. Additionally, IRES‐dependent translation initiation relies on the lineage‐specific IRES trans‐acting factors (ITAFs) [49]. ITAFs exert RNA chaperone activity by restructuring IRES RNA conformations, thereby maximizing the efficiency of intermolecular contacts between IRES sequences and core components of the translation machinery [44]. Here, we found that mutations affect the secondary structure of the IRES, thereby influencing the translation efficiency. However, we did not further investigate whether the two mutations in the IRES impact the regulatory effects of eIFs and ITAFs on IRES, as well as the efficiency of ribosome binding to IRES. This will be the focus of our future research. We observed that in the SVA IRES, although the mutation at the 5^′^ end did not cause significant changes in the structural domain, it still had a notable impact on the virus’s growth kinetics, indicating that the 5^′^ end sequence of the IRES is critically important. Furthermore, base mutations in domain II may induce secondary structural changes within the domain, which can also significantly affect viral growth kinetics. These findings have not been reported previously.

Viruses must rely on host cells for their replication. Consequently, they have developed complex mechanisms to interfere with the host’s protein synthesis system, so that viral proteins are produced first. This interference not only reduces the competition for translational resources but also inhibits the host’s innate immune mechanisms [57]. The IRES mutations identified in this study were obtained through serial passaging under strong innate immune selection. Thus, these mutations probably help the virus to evade the innate antiviral immune response. Further analysis showed that the mutant viruses did not evade host innate immunity; on the contrary, they induced a stronger antiviral innate immune response than the WT virus following infection. Thus, the data support the conclusion that these mutations enhance viral fitness by boosting IRES‐driven translation rather than escaping innate immunity. Interestingly, the IRES‐108U109 mutation is absent from all reported SVA strains, whereas the IRES‐G265A mutation is highly conserved in most strains. These findings indicate that both mutations confer advantages to SVA; however, the IRES‐G265A mutation arises more readily during natural evolution, suggesting that all SVA lineages may eventually converge on this genotype. In contrast, the IRES‐108U109 mutation appears difficult to attain in nature, making it unlikely that SVA strains will evolve toward this particular sequence.

Prior research has confirmed that viruses develop adaptive mutations (including immune escape and replication‐enhancing mutations), which create fitness trade‐offs. Such mutations help viruses survive and replicate more effectively under specific selective pressures, such as host innate immunity. However, they weaken the virus’s fundamental capacity for replication, protein production, and transmission when immune pressure is absent. The natural loss of viral function caused by these mutations is defined as fitness cost [58, 59]. In this study, we identified IRES mutations after 80 serial viral passages under strong innate antiviral immune pressure. However, the IRES‐108U109 mutation is barely detected in global wild SVA isolates; this indicates that the mutation brings a large fitness cost. Without persistent strong immune selection in natural environments, the mutation cannot be stably maintained in viral populations. In contrast, the IRES‐G265A mutation is highly conserved among field strains, demonstrating that this substitution imposes little‐to‐no fitness cost on the virus.

In summary, we performed up to 80 serial passages of SVA in cell lines with enhanced antiviral innate immune pressure. We observed that CRL‐FJ carried far more nucleotide mutations than IB‐FJ. These findings demonstrate that increased antiviral innate immune pressure elevates viral mutation rates and serves as a major driver of viral evolution. Furthermore, we discovered that even single‐nucleotide substitutions within the IRES region of CRL‐FJ markedly boost protein translation efficiency, conferring a replicative advantage to the virus in host cells. Notably, our data indicate that these IRES mutations do not promote viral replication by improving the virus’s ability to evade innate antiviral immunity.

**Nomenclature**
SVA: Senecavirus A
UTR: Untranslated region
IRES: Internal ribosome entry site
CCM: Conditioned medium from SVA infected CRL‐2843 cells
WT: Wild type
Mut: Mutations
MOI: Multiplicity of infection
Fluc: Firefly luciferase
Rluc: Renilla luciferase
USA: United States of America.

## Author Contributions

**Tao Li**: methodology, investigation, formal analysis, data curation, writing – original draft. **Wen Dang**: project administration, methodology, resources, validation, supervision. **Weiwei Li**: resources, supervision, writing – review and editing. **Xiaodong Qin**: resources, supervision, writing – review and editing. **Fan Xu**: formal analysis. **Hong Tian**, **Fan Yang, and Yanyan Chang:** resources. **Haixue Zheng:** conceptualization, methodology, resources, supervision, funding acquisition.

## Funding

This study was funded by the Key Project of National Natural Science Foundation of China (Grant 32330107), Project of National Center of Technology Innovation for Pigs (Grant NCTIP‐XD/C03), and Innovation Program of Chinese Academy of Agricultural Sciences (Grants CAAS‐BRC‐202608 and CAAS‐ASTIP‐2026‐LVRI).

## Disclosure

AI‐assisted content was rigorously reviewed and revised by the authors to ensure factual accuracy, originality, and adherence to academic integrity.

## Ethics Statement

No animal experiments were conducted in this study. All experiments involving SVA were conducted in the Biosafety Level 2 (BSL‐2) facilities of Lanzhou Veterinary Research Institute, Chinese Academy of Agricultural Sciences, and approved by the Ministry of Agriculture and Rural Affairs of China.

## Conflicts of Interest

The authors declare no conflicts of interest.

## Data Availability

The data that support the findings of this study are available from the authors upon reasonable request.
